# Programa para Otimizar a Detecção da Fibrilação Atrial Paroxística: Estudo Ritmo

**DOI:** 10.36660/abc.20240235

**Published:** 2024-09-17

**Authors:** Rodrigo Paashaus de Andrade, Priscila Valverde Oliveira Vitorino, Ana Luiza Lima Sousa, Roberto Dischinger Miranda, Bruno Augusto Alcova Nogueira, Elizabeth do Espírito Santo Cestário, Marcus Vinícius de Oliveira, Luiz Kencis, Fernando Cenci Tormen, Pablo de Oliveira Antunes, Ivan Di Beo, Luiz Eduardo Guiselli Gallina, Weimar Kunz Sebba Barroso

**Affiliations:** 1 Universidade Federal de Goiás Faculdade de Medicina Goiânia GO Brasil Programa de Pós-graduação em Ciências da Saúde - Faculdade de Medicina - Universidade Federal de Goiás, Goiânia, GO – Brasil; 2 Pontifícia Universidade Católica de Goiás Escola de Ciências Sociais e da Saúde Goiânia GO Brasil Programa de Pós-graduação em Atenção à Saúde - Escola de Ciências Sociais e da Saúde - Pontifícia Universidade Católica de Goiás, Goiânia, GO – Brasil; 3 Universidade Federal de Goiás Unidade de Hipertensão Arterial Goiânia GO Brasil Unidade de Hipertensão Arterial - Universidade Federal de Goiás, Goiânia, GO – Brasil; 4 Universidade Federal de São Paulo Disciplina de Geriatria e Gerontologia São Paulo SP Brasil Serviço de Cardiologia, Disciplina de Geriatria e Gerontologia - Universidade Federal de São Paulo, São Paulo, SP – Brasil; 5 Clínica Coração Vivo São José dos Campos SP Brasil Clínica Coração Vivo, São José dos Campos, SP – Brasil; 6 Clínica Cardiológica e UNIFEV Votuporanga SP Brasil Clínica Cardiológica e UNIFEV, Votuporanga, SP – Brasil; 7 Cardiodiagnósticos Goiânia GO Brasil Cardiodiagnósticos, Goiânia, GO – Brasil; 8 Lapacor São Paulo SP Brasil Lapacor, São Paulo, SP – Brasil; 9 Clínica Cardiologic Bento Gonçalves RS Brasil Clínica Cardiologic, Bento Gonçalves, RS – Brasil; 10 Instituto Médico Tiaminho Daikura Águas de Lindóia SP Brasil Instituto Médico Tiaminho Daikura, Águas de Lindóia, SP – Brasil; 11 Climed Clínica Médica Peruíbe SP Brasil Climed Clínica Médica, Peruíbe, SP – Brasil; 12 Clínica Cuore Arapongas PR Brasil Clínica Cuore, Arapongas, PR – Brasil; 13 Hospital Albert Einstein Goiânia GO Brasil Hospital Albert Einstein, Goiânia, GO – Brasil

**Keywords:** Fibrilação Atrial, Hipertensão, Insuficiência Cardíaca, Eletrocardiografia

## Abstract

**Fundamento:**

A fibrilação atrial (FA) é a arritmia cardíaca sustentada mais frequente, mas ainda é subdiagnosticada especialmente em pacientes assintomáticos.

**Objetivo:**

Avaliar uma estratégia simples para otimizar a identificação da FA.

**Métodos:**

Avaliados indivíduos assintomáticos com 65 anos ou mais, portadores de hipertensão arterial (HA) ou insuficiência cardíaca (IC). Os dados foram inseridos e armazenados em plataforma REDCap. Inicialmente foram realizadas análise de risco de FA com o algoritmo matemático *Stroke Risk Analysis* (SRA) aplicado em eletrocardiograma (ECG) de 1 hora. Todos os pacientes de alto risco de FA foram orientados a realizar o protocolo de ECG domiciliar por sete dias com o equipamento portátil Kardia 6L OMRON, AliveCor^®^. O teste de Kolmogorov-Smirnov foi usado para verificar a normalidade da distribuição das variáveis quantitativas; aquelas com distribuição normal foram expressas em média e desvio-padrão. Adotou-se como significativo o valor de p<0,05.

**Resultados:**

Foram avaliados 423 pacientes; 15 foram excluídos por não terem realizado o SRA, resultando em uma amostra de 408 pacientes. A avaliação evidenciou que 13 (3,2%) pacientes apresentaram FA, 120 (29,4%) foram considerados de alto risco para FA e 275 (67,4%) sem risco aumentado. Dos 120 pacientes de alto risco, 111 realizaram adequadamente o protocolo de sete dias com o Kardia, tendo sido identificados um ou mais registros de FA em 43 pacientes.

**Conclusão:**

A estratégia adotada no estudo RITMO mostrou-se eficaz para identificar, com uma incidência de 13,7% (56/408), episódios de FA em pacientes idosos assintomáticos e portadores de HA ou IC.

## Graphical abstract

**Figure:**
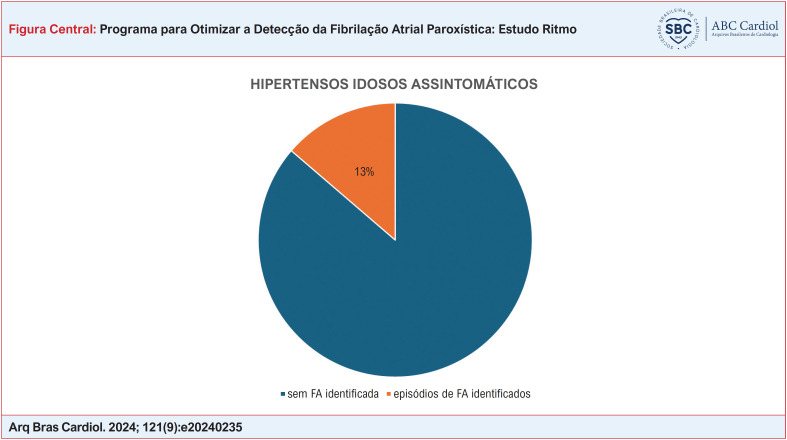

## Introdução

A fibrilação atrial (FA) é a arritmia cardíaca sustentada mais frequente em adultos, com uma prevalência estimada em 2-4%, aumentando progressivamente com a idade. O número de episódios subdiagnosticados é alto, especialmente na forma paroxística. Estima-se, nas próximas décadas, um aumento de 2,3 vezes nessa prevalência em razão do envelhecimento da população e do aprimoramento e otimização dos métodos diagnósticos.^1-3^

A FA pode acontecer na forma assintomática ou oligossintomática, o que dificulta a identificação e potencializa o risco de eventos tromboembólicos associados. É importante, portanto, estar atento aos pacientes portadores de fatores de risco e considerar a adoção de estratégias mais efetivas para o rastreamento e identificação dessa arritmia.^4-6^ Dentre os diversos fatores de risco para o desenvolvimento da FA, destacam-se idade avançada, hipertensão arterial (HA), insuficiência cardíaca (IC) e doença arterial coronariana.^4,5^

Na suspeita de FA, os métodos complementares mais utilizados para tentar estabelecer o diagnóstico são o eletrocardiograma (ECG) de 12 derivações e o Holter de 24 horas. Entretanto, esses métodos podem não identificar mais de 50% dos casos, principalmente na forma paroxística.^7^ Por outro lado, estratégias como o Holter de longa duração, ECG de uso domiciliar, algoritmos baseados em variabilidade de RR e outros sistemas vestíveis que se propõem a aumentar essa capacidade de identificação, especialmente da forma paroxística, têm sido objeto de estudos clínicos.^4,5,7-9^

Frente a esse desafio, foi desenvolvido e executado o "Programa para Otimizar a Detecção da FA Paroxística", com o objetivo de avaliar uma estratégia simples e acessível para otimizar a identificação dessa arritmia em idosos assintomáticos portadores de HA ou IC.

## Métodos

### Caracterização do estudo

Estudo transversal descritivo, realizado com indivíduos assintomáticos, ≥65 anos, portadores de HA ou IC, no Hospital das Clínicas da Universidade Federal de Goiás (UFG), por um centro coordenador e coinvestigadores cardiologistas que participaram do treinamento e coleta de dados.

O estudo foi aprovado pelo Comitê de Ética em Pesquisa do Hospital das Clínicas da UFG sob o nº CAE 58646322.9.0000.5078. Todos os participantes assinaram o Termo de Consentimento Livre e Esclarecido, antes do início do estudo.

### População estudada

Para o cálculo da população amostral, considerou-se a população brasileira de idosos e a prevalência de FA nessa faixa etária de 3,8%. Foram utilizados o nível e o intervalo de confiança de 95% e 5%, respectivamente, obtendo-se uma amostra de 385 participantes.^10,11^

Os pacientes foram selecionados nos serviços de Cardiologia e convidados a participar no estudo. A fase de inclusão foi de maio de 2023 até dezembro de 2023 e cada paciente recebeu um número de identificação no estudo, composto de quatro dígitos: os dois primeiros referentes ao local de coleta e os dois últimos ao número do paciente.

### Critérios de inclusão

Foram incluídos no estudo idosos com ≥65 anos, que apresentavam diagnóstico clínico de HA ou de IC, tendo como base a história médica, a revisão do prontuário e as medicações em uso.

### Critérios de exclusão

Pacientes que apresentassem diagnóstico prévio de FA, identificado por meio do prontuário, ECG ou Holter, ou que estivessem participando de outros protocolos de pesquisa.

### Procedimentos do estudo

Os dados do estudo foram coletados e gerenciados usando o REDCap (*Research Electronic Data Capture*), hospedado na UFG. O REDCap é uma plataforma de *software* segura, baseada na *web* e projetada para apoiar a captura de dados para pesquisa, fornecendo: uma interface intuitiva para captura de dados validados; trilhas de auditoria para rastrear manipulação de dados e procedimentos de exportação; procedimentos automatizados de exportação para *downloads* contínuos de dados para pacotes estatísticos comuns; e procedimentos para a integração de dados e interoperabilidade com fontes externas.^12,13^

Cada paciente foi cadastrado no endereço eletrônico <www.sra.cardios.net>, com preenchimento de dados para a realização do *Stroke Risk Analysis* (SRA) por meio do *software* CardioNet. A realização do SRA ocorreu com o equipamento Cardio Light^®^ (Cardios, São Paulo, Brasil) e registro eletrocardiográfico durante 1 hora. Durante esse período, o paciente se mantinha no ambiente ambulatorial e poderia realizar as atividades usuais, desde que não desconectasse o circuito. A localização dos eletrodos seguiu a técnica padronizada para a realização do ECG com tricotomia prévia. Ao final do teste, os dados eram transferidos do dispositivo portátil para a plataforma do SRA.

**Figura 1:**
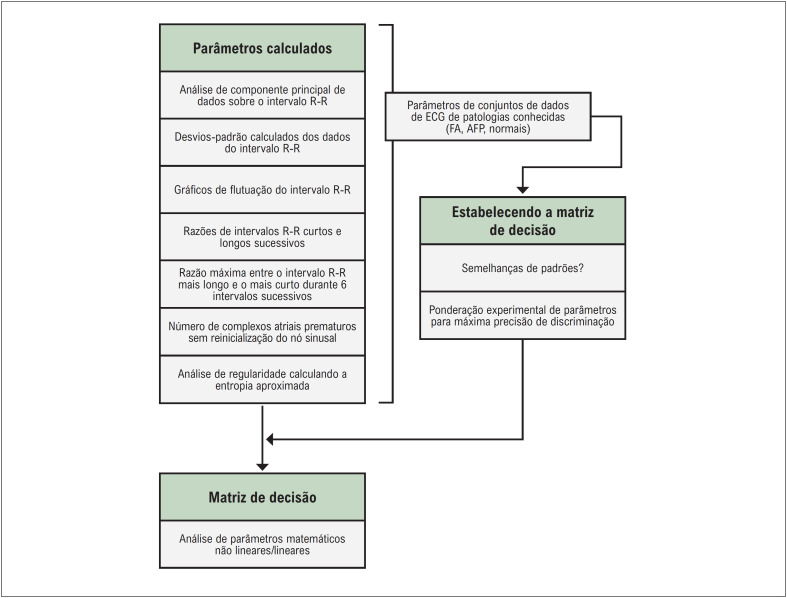
Parâmetros utilizados na matriz de decisão do algoritmo SRA. SRA: Stroke Risk Analysis; ECG: eletrocardiograma; FA: fibrilação atrial. Fonte: Duning et al.^14^

O algoritmo matemático SRA avalia vários parâmetros de variabilidade de RR no ECG de uma hora e consegue entregar três possíveis cenários como matriz de decisão derivada do gráfico de Poincaré: sem risco aumentado de FA, risco alto de FA e presença de FA no registro de uma hora (Figura 1).^14,15^

Após a realização do SRA, os pacientes sem risco aumentado continuaram seu acompanhamento médico conforme a rotina de cada serviço. Aqueles que tiveram FA identificada no registro de uma hora receberam orientação adequada para o tratamento e acompanhamento da arritmia; e aqueles que apresentaram o resultado de alto risco para FA foram selecionados para a realização do ECG domiciliar.

**Figura 2:**
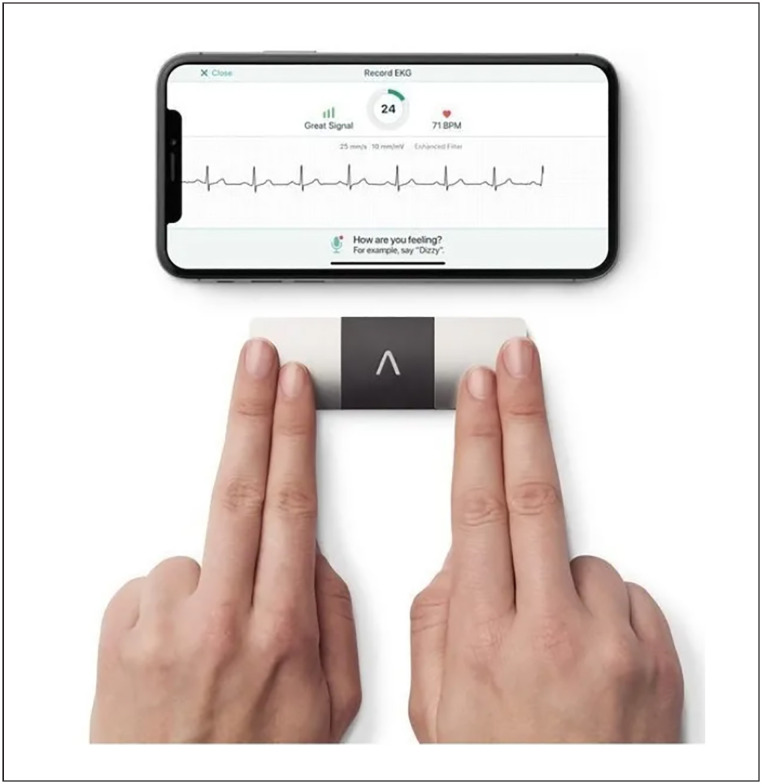
Aparelho de eletrocardiograma portátil de seis derivações (Kardia 6L); fonte: OMRON.

O ECG domiciliar foi realizado com o equipamento portátil Kardia 6L OMRON/AliveCor^®^, que registra seis derivações, durante 30 segundos. Inicialmente, o aplicativo (app) para a conexão e *download* do registro eletrocardiográfico foi instalado pelo coinvestigador no aparelho celular do próprio paciente. Em seguida, os participantes foram orientados a realizar três registros diários (manhã, tarde e noite, em horário aleatório) e registros adicionais em caso de sintomas de palpitações, por sete dias consecutivos. O primeiro registro foi realizado pelo cardiologista, com as orientações para o paciente e/ou cuidador (Figura 2).

**Figura 3:**
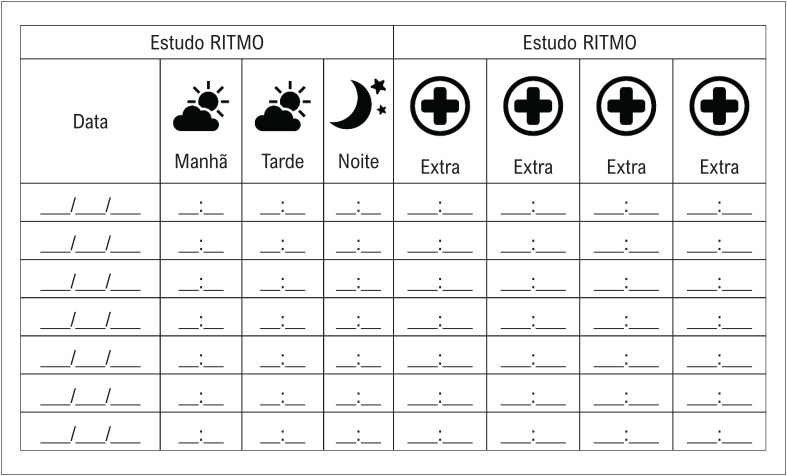
Diário para anotar os registros eletrocardiográficos.

Para aumentar a adesão do participante ao protocolo de monitorização domiciliar do ECG, um diário de registro foi dispensado com a orientação adequada quanto à realização, preenchimento do dia e do horário (Figura 3).

Para os pacientes com SRA de alto risco que não retornaram para realizar o ECG domiciliar, foi considerado, para fins de análise, ECG sem presença de FA.

Ao final de uma semana, o paciente retornava ao serviço para a entrega do equipamento e orientações relacionadas aos resultados. Todos os registros eletrocardiográficos eram transmitidos via app para uma plataforma central com acesso pelo centro coordenador e analisado por algoritmos validados para identificação da arritmia.

### Instrumentos e variáveis do estudo

O estudo utilizou três instrumentos: um questionário sociodemográfico e clínico; o relatório do SRA disponibilizado no próprio *software* e o relatório do ECG domiciliar (Tabela 1).

**Tabela 1:** Instrumentos, variáveis, unidades de medida e categorias

Instrumentos	Variáveis	Unidades de medida ou categorias
Questionário sociodemográfico e clínico	Iniciais e número de registro	Utilizado para organização do estudo
	Sexo	Masculino
		Feminino
	Idade	Calculada em anos a partir da data de nascimento
	Estado civil	Solteiro(a)
		Casado(a)
		Divorciado(a)
		Viúvo(a)
	Escolaridade	Analfabeto
		Ensino fundamental completo
		Ensino fundamental incompleto
		Ensino médio completo
		Ensino médio incompleto
		Ensino superior completo
		Ensino superior incompleto
		Pós-graduação completa
		Pós-graduação incompleta
	Tabagismo	Atual
		Ex-tabagista
		Não
	Consumo de bebida alcóolica	Não consome
		Dentro do recomendado
		Acima do recomendado
	Atividade física	Ativo (acima de 150 min por semana)
		Sedentário (abaixo de 150 min por semana)
	Diagnóstico clínico	Hipertensão arterial
		Insuficiência cardíaca
SRA	Resultado SRA	Presença de FA
		Risco aumentado de FA
		Sem risco aumentado para FA
ECG	Presença ou ausência de FA em qualquer ECG dos sete dias	Presença de FA
		Ausência de FA

SRA: Stroke Risk Analysis; ECG: eletrocardiograma; FA: fibrilação atrial.

### Análise estatística

Os dados foram exportados do RedCap para análise estatística no Jamovi 2.2.5. Foi realizada análise descritiva com a utilização de medidas de tendência central e dispersão para variáveis quantitativas e frequência para as qualitativas. O teste de Kolmogorov-Smirnov foi aplicado para verificar a normalidade da distribuição das variáveis quantitativas; as que apresentaram distribuição normal foram expressas em média ± desvio-padrão. Adotou-se como significativo o valor de p<0,05.

## Resultados

Foram avaliados 423 pacientes. Destes, 15 foram excluídos por SRA não realizado, resultando em uma amostra de 408 pacientes. A idade média foi 75,2 ± 7,3 anos, o Índice de Massa Corporal (IMC) médio foi 27,3±7,3 Kg/m^2^ e 66,4% eram do sexo feminino. O diagnóstico de HA foi o critério de inclusão mais frequente, com 96,3% da população (Tabela 2).

**Tabela 2:** Caracterização da população estudada

Variáveis		n (%)
**Estado civil**		
	Com companheiro(a)	243 (59,5)
	Sem companheiro	162 (39,8)
	Sem informação	3 (0,7)
**Escolaridade**		
	Analfabeto	24 (5,9)
	Ensino fundamental (incompleto ou completo)	199 (48,8)
	Ensino médio (incompleto ou completo)	101 (24,8)
	Ensino superior (incompleto ou mais)	84 (20,6)
**Tabagista**		16 (4,0)
**Consumo de bebida alcóolica**		68 (16,6)
**Sedentarismo**		209 (51,4)
**Número de classes de anti-hipertensivos**		
	Uma	136 (35,0)
	Duas	135 (34,7)
	Três ou mais	118 (30,3)
**Classes de anti-hipertensivos**		
	Inibidores da enzima de conversão da angiotensina	69 (16,9)
	Bloqueadores dos receptores AT1 da angiotensina II	247 (60,5)
	Bloqueadores dos canais de cálcio	142 (34,8)
	Betabloqueadores	158 (38,7)
	Diuréticos	159 (39,0)
	Outros	16 (3,9)
**Estatinas**		269 (65,9)
**Antidiabéticos orais**		107 (26,2)

Em relação aos 408 pacientes que realizaram o SRA, foram observados os seguintes achados: 13 (3,2%) apresentaram FA, 120 (29,4%) foram considerados de alto risco para FA e 275 (67,4%) sem risco aumentado.

**Figura 4:**
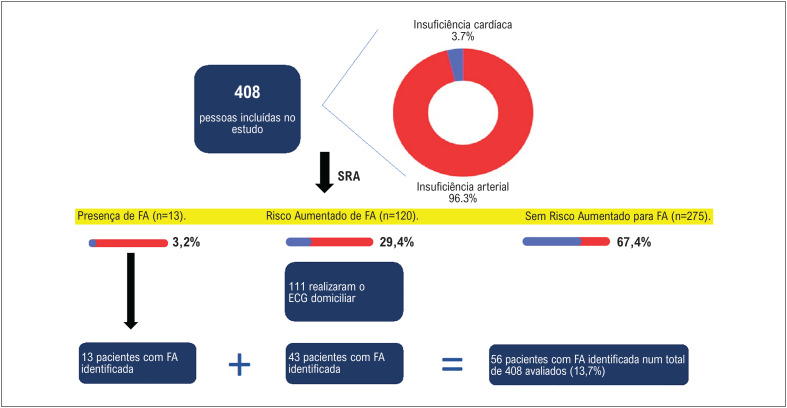
Achados de FA pelo SRA e pelo KARDIA na população estudada. SRA: Stroke Risk Analysis; FA: fibrilação atrial.

Dos 120 pacientes de alto risco, 111 realizaram adequadamente o protocolo de sete dias com o Kardia; nove (7,5%) pacientes não retornaram para receber as orientações e o aparelho para a ECG domiciliar, tendo sido considerado, por convenção do protocolo, como ausência de FA. Foram identificados um ou mais registros de FA pelo Kardia, durante os sete dias de acompanhamento, em 43 pacientes (Figura 4).

Avaliou-se também a frequência do diagnóstico de FA pelo SRA e pelo ECG domiciliar nos diferentes diagnósticos de HA e IC (Tabela 3).

**Tabela 3:** Fibrilação atrial diagnosticada pelo SRA, pelo ECG domiciliar e total, segundo o diagnóstico clínico

Diagnósticos clínico	FA no SRA n (%)	FA no ECG n (%)	FA total n (%)
Hipertensão arterial (n=393)	11 (2,8)	40 (10,2)	51 (13,0)
Insuficiência cardíaca (n=15)	2 (13,0)	3 (20,0)	5 (33,3)
Total (n=408)	13 (3,2)	43 (10,5)	56 (13,7)

SRA: Stroke Risk Analysis; ECG: eletrocardiograma; FA: fibrilação atrial.

## Discussão

A prevalência estimada de FA na população adulta é de 2-4%, mas em idosos, hipertensos e portadores de IC, essa taxa é seguramente maior. A FA é responsável por 20-30% dos acidentes vasculares cerebrais (AVC) isquêmicos e 10% dos criptogênicos, e esse risco é duas vezes maior em portadores de HA ou IC e uma vez e meia maior nos idosos. Apesar de assintomáticos, idosos com HA ou IC apresentam risco mais alto tanto para a FA quanto para as complicações a ela relacionadas.^16,17^

Neste estudo, dos 423 pacientes inicialmente convidados a participar, apenas 15 (3,5%) não realizaram o SRA, o que evidencia uma baixa resistência para a realização do método. Esse fato coloca o SRA como uma alternativa plausível em caso de um Holter de 24 horas normal ao investigar a possibilidade de arritmia, pois, como se sabe, 50% dos episódios não são identificados por esse método. Além disso o método SRA tem sensibilidade e especificidade acima de 95% para identificar risco elevado de FA na comparação com o Holter de 24 horas.^15,18^

Vale ressaltar que 96,3% da população estudada foi constituída por idosos com HA e, portanto, os achados devem ser discutidos especialmente dentro desse contexto. Por outro lado, trata-se de população muito frequente na rotina dos atendimentos médicos, sugerindo que os resultados obtidos possam ser de grande utilidade na prática clínica.^19^

Todos os pacientes avaliados estavam em uso de anti-hipertensivos, sendo a classe dos bloqueadores dos receptores de angiotensina a mais frequente (60,5%) e o uso de anti-hipertensivos em combinações correspondeu a 65% do total. Ainda, o uso de estatinas e antidiabéticos orais aconteceu em 65,9% e 26,2% dos pacientes, respectivamente, caracterizando a elevada prevalência de fatores de risco como a dislipidemia e diabetes nessa população.^20,21^

Destaca-se, na amostra avaliada, a elevada prevalência de alto risco para a FA (29,4%) além dos 3,2% com a arritmia já identificada durante o registro de 1 hora para o SRA. Como se sabe, o risco cumulativo para a FA aumenta de forma significativa após os 65 anos, mas identificar essa arritmia em indivíduos assintomáticos ainda é considerado um grande desafio.^2,4,22^

Dos 120 pacientes identificados como de alto risco pelo SRA, 111 realizaram a eletrocardiografia domiciliar por sete dias com o Kardia 6 derivações. Foram identificados episódios de FA em 43 pacientes, ou seja, cerca de 1/3 dos pacientes de alto risco pelo SRA apresentou um ou mais episódios de FA identificados no domicílio. No estudo VITAL-AF^23^ foram avaliados 30 715 pacientes (atendidos na atenção primária) com mais de 65 anos e sem diagnóstico prévio de FA; metade da amostra foi randomizada para realizar o Kardia de uma derivação no momento da consulta e foram identificados novos episódios de FA em 1,72% e 1,59% no grupo *screening* e no grupo-controle (p=0,38), respectivamente.^23^ Os achados do estudo VITAL-AF não demonstraram efetividade em adicionar o uso do Kardia de uma derivação durante a avaliação na atenção primária na comparação com o grupo-controle; por outro lado, os achados do presente estudo indicaram maior efetividade da monitorização domiciliar com o Kardia, selecionando-se previamente, por meio da SRA, aqueles de alto risco para a FA. Em outro estudo, com amostra constituída por pacientes com história prévia de FA revertida, ou seja, de alto risco para novos episódios, o uso domiciliar do Kardia 6 derivações mostrou-se uma estratégia eficaz para a detecção precoce da arritmia.^24^

Em 408 pacientes, foram identificados episódios de FA tanto pelo SRA (3,2%) quanto pelo Kardia (10,5%) (Figura 4). Quando se avaliou a frequência de FA nos pacientes hipertensos e naqueles com IC, foram encontrados valores de 13% e 33,3%, respectivamente, mas esse dado deve ser analisado com cautela pois apenas 3,7% da população apresentava IC.

### Limitações do estudo

Os resultados encontrados neste estudo referem-se quase na totalidade a idosos hipertensos e, portanto, há que se ter cautela na análise dos dados relacionados aos idosos com IC. Por outro lado, chama a atenção a incidência ainda mais alta de FA nos pacientes com IC e remete à necessidade de estudos específicos para essa doença. Os pacientes sem risco aumentado para FA pelo SRA não realizaram o ECG domiciliar, o que impossibilita a análise da incidência da arritmia nesse subgrupo do estudo. A incidência de FA identificada no estudo RITMO referiu-se à análise automatizada do algoritmo AliveCor Kardia, mas é sempre desejável a revisão e validação da arritmia por profissional qualificado. Ainda, vale considerar que alguns pacientes podem ter apresentado episódios de FA em momentos em que não houve o registro eletrocardiográfico com o KARDIA, ou seja, a incidência pode ser ainda mais alta do que a descrita com essa estratégia.

## Conclusão

A estratégia adotada no estudo RITMO mostrou-se eficaz para identificar episódios de FA em pacientes idosos assintomáticos e portadores de HA ou IC.
