# Supplementary material for: Programa para Otimizar a Detecção da Fibrilação Atrial Paroxística: Estudo Ritmo
Source: Arq Bras Cardiol. 2024 Sep 17;121(9):e20240235. [Article in Portuguese] doi: 10.36660/abc.20240235 (PMC11495592; doi:10.36660/abc.20240235)
Supplement: Translation

# Translation: A Program to Optimize the Detection of Paroxysmal Atrial Fibrillation: The RITMO Study

**DOI:** 10.36660/abc.20240235i

**Authors:** Rodrigo Paashaus de Andrade, Priscila Valverde Oliveira Vitorino, Ana Luiza Lima Sousa, Roberto Dischinger Miranda, Bruno Augusto Alcova Nogueira, Elizabeth do Espírito Santo Cestário, Marcus Vinícius de Oliveira, Luiz Kencis, Fernando Cenci Tormen, Pablo de Oliveira Antunes, Ivan Di Beo, Luiz Eduardo Guiselli Gallina, Weimar Kunz Sebba Barroso

**Affiliations:** 1 Goiânia GO Brazil Programa de Pós-graduação em Ciências da Saúde - Faculdade de Medicina - Universidade Federal de Goiás, Goiânia, GO – Brazil; 2 Goiânia GO Brazil Programa de Pós-graduação em Atenção à Saúde - Escola de Ciências Sociais e da Saúde - Pontifícia Universidade Católica de Goiás, Goiânia, GO – Brazil; 3 Goiânia GO Brazil Unidade de Hipertensão Arterial - Universidade Federal de Goiás, Goiânia, GO – Brazil; 4 São Paulo SP Brazil Serviço de Cardiologia, Disciplina de Geriatria e Gerontologia - Universidade Federal de São Paulo, São Paulo, SP – Brazil; 5 São José dos Campos SP Brazil Clínica Coração Vivo, São José dos Campos, SP – Brazil; 6 Votuporanga SP Brazil Clínica Cardiológica e UNIFEV, Votuporanga, SP – Brazil; 7 Goiânia GO Brazil Cardiodiagnósticos, Goiânia, GO – Brazil; 8 São Paulo SP Brazil Lapacor, São Paulo, SP – Brazil; 9 Bento Gonçalves RS Brazil Clínica Cardiologic, Bento Gonçalves, RS – Brazil; 10 Águas de Lindóia SP Brazil Instituto Médico Tiaminho Daikura, Águas de Lindóia, SP – Brazil; 11 Peruíbe SP Brazil Climed Clínica Médica, Peruíbe, SP – Brazil; 12 Arapongas PR Brazil Clínica Cuore, Arapongas, PR – Brazil; 13 Goiânia GO Brazil Hospital Albert Einstein, Goiânia, GO – Brazil

**Keywords:** Atrial Fibrillation, Hypertension, Heart Failure, Electrocardiography

## Abstract

**Background:**

Atrial fibrillation (AF) is the most common sustained arrythmia, but still underdiagnosed especially among asymptomatic patients.

**Objectives:**

To evaluate a simple strategy to optimize the identification of AF.

**Methods:**

Asymptomatic patients aged 65 years or older, with hypertension or heart failure (HF), were included. Data were inserted into the REDCap software platform. Patients were assessed for the risk for AF using the Stroke Risk Analysis (SRA) mathematical algorithm, which was applied on a one-hour electrocardiogram (ECG). All patients at high risk for AF were instructed to follow a home ECG protocol for seven days using a portable Kardia 6 (OMRON, AliveCor^®^). The Kolmogorov-test was used to test the normality of quantitative variables; those with normal distribution were expressed as mean and standard deviation. A p<0.05 was set as statistically significant.

**Results:**

A total of 423 patients were assessed; 15 were excluded due to absence of SRA, yielding a sample of 408 patients. In 13 (3.2%), AF was identified, 120 (29.4%) were considered at high risk and 275 (67.4%) without increased risk for AF. Of the 120 high-risk patients, 111 successfully completed the seven-day protocol with Kardia; at least one episode of AF was identified in 43 patients.

**Conclusion:**

The strategy adopted in the RITMO study was shown to be effective in identifying AF in asymptomatic elderly patients with hypertension or HF, with an incidence of 13.7% (56/408).

## Introduction

Atrial fibrillation (AF) is the most common sustained arrythmia in adults, with an estimated prevalence of 2-4%, which increases with age. The number of underdiagnosed cases, especially paroxysmal AF, is large. It is expected an increase in this prevalence by 2.3 times in the next decades, due to population aging and optimization of diagnostic methods.^1-3^

Patients with FA may be asymptomatic or oligosymptomatic, which complicates the identification of the disease and increases the risk of thromboembolic events. Therefore, it is important to be alert to patients with risk factors and consider the adoption of more effective strategies to screen for and identify this arrhythmia.^4-6^ Advanced age, hypertension, heart failure (HF), and coronary artery disease are among the main risk factors for AF.^4,5^

In suspected AF, the most used complementary tests in attempt to confirm the diagnosis are the 12-lead electrocardiogram (ECG) and 24-hour Holter monitoring. However, more than 50% of the cases may not be diagnosed by these tests, particularly paroxysmal AF.^7^ On the other hand, long-term Holter monitoring, home ECG monitoring, algorithms based on RR variability and other wearable systems aiming at increasing diagnostic capacity, especially of paroxysmal FA, have been the subject of clinical trials.^4,5,7-9^

In light of this challenge, a "Program to optimize the detection of paroxysmal AF" was created and implemented, aiming at evaluating a simple and accessible strategy to optimize the identification of this arrhythmia in asymptomatic elderly patients with hypertension or HF.

## Methods

### Characteristics of the study

This was a descriptive cross-sectional study conducted with asymptomatic patients aged ≥ 65 years, with hypertension or HF by a coordinating center and coinvestigators (cardiologists) that participated in the training and data collection. The study was carried out at the general hospital of the Federal University of Goias.

The study was approved by the ethics committee of the hospital (approval number 58646322.9.0000.5078). All participants signed an informed consent form before the study was initiated.

### Study population

For sample size calculation, we considered the elderly population in Brazil and the prevalence of AF of 3.8% in this group. A 5% level and 95% interval of confidence was used, yielding a sample of 385 participants.^10,11^

Eligible patients were selected at the cardiology division who were invited to participate in the study. Patients were included from May to December 2023; each patient was assigned an identification number composed of four digits: the first two referring to the place of collection and the last two referring to the patient identification number.

### Inclusion criteria

Patients aged 65 years or older, with diagnosis of hypertension or HF based on medical history, review of medical records and medications used were included in the study.

### Exclusion criteria

Patients with previous diagnosis of AF identified from the medical records or by ECG or Holter, and patients participating in other study protocols were excluded.

### Study procedures

Data were collected and managed using the Research Electronic Data Capture (REDCap) hosted at the Federal University of Goias. REDCap is a safe, web-based software platform, designed to support data capture for research studies. REDCap provides an intuitive interface to capture validated data; audit trail to detect data manipulation and exportation procedures; automated data export functions continuous downloads to common statistical packages; and procedures for data integration and interoperability with external sources.^12,13^

Each patient was registered on <www.sra.cardios.net>, with insertion of data for the Stroke Risk Analysis (SRA) using the CardioNet software. Electrocardiographic recordings were performed for one hour using the Cardio Light ® (Cardios, Sao Paulo, Brazil) equipment for the SRA. During this period, the patient was free to move in an outpatient environment, while connected to this circuit. The electrodes were placed on standard positions used for an ECG after patient's hair removal from these sites. At the end of the test, data were transferred from the portable device to the SRA platform.

The SRA mathematical algorithm analyses parameters of RR variability on ECG, providing three different scenarios as decision matrix derived from the Poincaré plot: without increased risk for AF, high risk for AF, and presence of AF detected in the first hour of electrocardiographic recordings (Figure 1).^14,15^

**Figure 1:** Parameters used in the decision matrix of the stroke risk analysis; ECG: electrocardiogram; AF: atrial fibrillation; PAF: paroxysmal atrial fibrillation; source: Duning et al.^14^

After the SRA, patients without increased risk for AF continued follow-up according to the clinical routine at each clinic. Those with AF identified during the one-hour monitoring received instructions about treatment and monitoring of arrhythmia, and patients at high risk for AF were assigned to home ECG.

Home ECG recording was carried out using a portable device (Kardia 6L OMRON/AliveCor^®^) with six leads and 30-second recordings. The app for connection and download of the electrocardiographic recordings was installed on patient's smartphone by the coinvestigator. Patients were instructed to make three ECG recordings (one in the morning, one in the afternoon and one at night, at any time) daily and additional recordings (if symptoms like palpitations were present) for seven consecutive days. The first recording was made by the cardiologist, who gave instructions to the patient or caregiver (Figure 2).

**Figure 2:** Mobile six-lead electrocardiogram device (Kardia 6L); source: OMRON.

In order to increase patient's adhesion to the protocol of home ECG monitoring, each patient was given a daily record form and instructed about the correct way to make the ECG recordings and complete the time sheet (Figure 3).

**Figure 3:** Daily time sheet for electrocardiographic recordings.

For patients at high risk for AF according to the SRA that did not return to perform home ECG, an ECG without AF was considered for analysis.

At the end of one week, the patient went to the center to return the device and receive instructions on the ECG results. All electrocardiographic recordings were sent by app to a central platform accessed by the coordinating center and analyzed for the presence of arrhythmia using several algorithms.

### Study instruments and variables

Three instruments were used in the study: a sociodemographic and clinical questionnaire; the SRA report available in the software, and the home ECG time sheet (Table 1).

**Table 1:** Instruments, variables, units of measurement and categories

Instruments	Variables	Units of measurement and categories
Sociodemographic and clinical questionnaire	Initials and registration number	Used for organization of the study
	Sex	Male
		Female
	Age	Calculated from date of birth (in years)
	Marital status	Single
		Married
		Divorced
		Widow/er
	School attainment	Illiterate
		Primary education
		Some primary education
		Secondary education
		Some secondary education
		Tertiary education
		Some tertiary education
		Postgraduation
		Some postgraduation
	Smoking	Current smoker
		Former smoker
		No
	Alcohol consumption	No
		Within recommendations
		Above recommendations
	Physical activity	Active (> 150 min/week)
		Sedentary (<150 min /week)
	Clinical diagnosis	Hypertension
		Heart failure
SRA	SRA result	Presence of AF
		Increased risk for AF
		Without increased risk for AF
ECG	Presence or absence of AF in any ECG in seven days	Presence of AF
		Absence of AF

SRA: Stroke Risk Analysis; ECG: electrocardiogram; AF: atrial fibrillation.

### Statistical analysis

Data were exported from the RedCap for statistical analysis using Jamovi 2.2.5. Descriptive analysis of the data was performed using central tendency measures and dispersion for quantitative variables and frequency for qualitative variables. The Kolmogorov-test was used to test the normality of quantitative variables; those with normal distribution were expressed as mean and standard deviation. A p<0.05 was set as statistically significant.

## Results

A total of 423 patients were evaluated; 15 were excluded due to absence of SRA, resulting in a sample of 408 patients. Mean age was 75.2 ± 7.3 years, mean body mass index (BMI) was 27.3±7.3 Kg/m^2^ and 66.4% of patients were women. A diagnosis of hypertension was the main inclusion criteria (96.3%) (Table 2)

**Table 2:** Characteristics of the study population

Variables		n (%)
**Marital status**		
	With a companion	243 (59.5)
	No companion	162 (39.8)
	No information	3 (0.7)
**Educational attainment**		
	Illiterate	24 (5.9)
	Primary education (completed or not)	199 (48.8)
	Secondary education (completed or not)	101 (24.8)
	Tertiary (completed or not)	84 (20.6)
**Smoking**		16 (4.0)
**Alcohol consumption**		68 (16.6)
**Sedentary lifestyle**		209 (51.4)
**Number of antihypertensive drug classes**		
	One	136 (35.0)
	Two	135 (34.7)
	≥ Three	118 (30.3)
**Classes de antihypertensives**		
	Angiotensin converting enzyme inhibitors	69 (16.9)
	Angiotensin II receptor blockers	247 (60.5)
	Calcium channel blockers	142 (34.8)
	Betablockers	158 (38.7)
	Diuretics	159 (39.0)
	Others	16 (3.9)
**Statins**		269 (65.9)
**Oral antidiabetics**		107 (26.2)

The SRA was carried out in 408 patients, yielding the following results: 13 (3.2%) had AF, 120 (29.4%) were at high risk for AF and 275 (67.4%) without increased risk.

Of the 120 patients at high risk, 111 successfully completed the seven-day protocol with Kardia. Nine (7.5%) patients did not return to receive instructions and start home ECG, and were then considered without AF, as previously established in the protocol. During the seven-day follow-up, at least one AF was identified by Kardia in 43 patients (Figure 4).

**Figure 4:** Identification of atrial fibrillation (AF) by stroke risk analysis (SRA) and KARDIA in the study population.

In addition, the frequency of AF identified by SRA and by home ECG in patients with hypertension and HF was evaluated (Table 3).

**Table 3:** Atrial fibrillation diagnosed by stroke risk analysis (SRA), home electrocardiogram by diagnosis (hypertension and atrial fibrillation)

Diagnosis	AF by SRA n (%)	AF by ECG n (%)	Total n (%)
Hypertension (n=393)	11 (2.8)	40 (10.2)	51 (13.0)
Heart failure (n=15)	2 (13.0)	3 (20.0)	5 (33.3)
Total (n=408)	13 (3.2)	43 (10.5)	56 (13.7)

SRA: Stroke Risk Analysis; ECG: electrocardiogram; AF: atrial fibrillation.

## Discussion

The prevalence of AF in adults is estimated at 2-4%, and in elderly hypertensive individuals and HF patients this prevalence is certainly higher. AF accounts for 20-30% of ischemic and 10% of cryptogenic cerebrovascular accidents; the risk is twice as high for patients with hypertension or HF and 1.5 fold higher among the elderly. Despite asymptomatic, older patients with hypertension or HF are at higher risk for both AF and its related complications.^16,17^

In the present study, of 423 patients who agreed to participate in the study, only 15 (3.5%) did not perform the SRA, indicating good compliance to the method. This suggests that the SRA is a plausible alternative in case of normal 24-hour Holter monitoring, since it is known that 50% of AF episodes are not detected by this method. Besides, as compared with 24-hour Holter, the SRA has a specificity and a sensitivity above 95% to identify an increased risk for AF.^15,18^

It is of note that 96.3% of the studied population was composed of older hypertensive patients and, therefore, our findings should be considered mainly within this context. On the other hand, this population is usually seen in medical practice, suggesting the usefulness of these findings in clinical practice.^19^

All patients were using antihypertensive drugs; angiotensin receptor blockers were the most commonly used (60.5%), and 65% of patients were taking antihypertensive combination treatment. Also, 65.9% and 26.2% of patients were using statins and oral antidiabetic agents, respectively, illustrating the high prevalence of risk factors like dyslipidemia and diabetes in this population.^20,21^

Also, in our sample, there was an increased prevalence of patients at high risk patients for AF (29.4%) in addition to those patients (3.2) with AF identified during the one-hour ECG monitoring for the SRA. Although it is known that the cumulative risk for AF significantly increases with age, to identify this arrhythmia in asymptomatic subjects is still challenging.^2,4,22^

Of the 120 patients at high risk for AF according to the SRA, 111 performed home ECG for seven days using six-lead Kardia. Episodes of AF were identified in 43 patients, *i.e*., nearly one third of the high-risk patients (identified by the SRA) showed at least one episode of AF detected by home. In the VITAL-AH^23^ study, 30,715 patients (seen in primary care clinics) older than 65 years old, without previous AF were evaluated; half of the patients were randomized to use a single-lead ECG (AliveCor KardiaMobile) during the visit and new episodes of AF were detected in 1.72% and 1.59% of the screening group and the control group (p=0.38) respectively.^23^ Based on these results, the use of the one-lead ECG Kardia device in primary care services was not effective as compared to the control group. On the other hand, our results indicated greater effectiveness of the home ECG using the Kardia device following previous identification of patients at higher risk for AF by the SRA. In another study, with patients with recurrent AF, the use of the KardiaMobile 6L was shown effective for early detection of arrythmia.^24^

In 408 patients, AF episodes were identified in 3.2% of patients by the SRA and n 10.5% of patients by the KardiaMobile 6L (Figure 4). The frequency of AF in patients with hypertension and in those with HF was 13% and 33.3%, respectively; however, these results should be interpreted with caution, since only 3.7% of the patients had HF.

### Study limitations

Almost all results of this study derived from older hypertensive patients and thus caution is needed to extrapolate them to older patients with HF. On the other hand, the higher incidence of AF in HF patients stands out and calls attention to the need for specific studies on the disease. Patients without increased risk for AF, as determined by the SRA, did not perform ECG, which made the analysis of the incidence of arrhythmia in this subgroup impossible. Although the incidence of AF reported in the RITMO study was determined by the automated analysis of the AliveCor Kardia algorithm, revision and validation of the presence of AF by a qualified professional is always desirable. Also, one should consider that some patients may have presented AF episodes at times with no electrocardiographic monitoring, *i.e*., the possibility that the incidence may have been underestimated by this strategy cannot be ruled out.

## Conclusion

The strategy adopted in the RITMO study was shown to be effective in identifying AF in asymptomatic elderly patients with hypertension or HF.
